# Identifying role of perceived quality and satisfaction on the utilization status of the community clinic services; Bangladesh context

**DOI:** 10.1186/s12913-016-1461-z

**Published:** 2016-06-24

**Authors:** Rizwanul M. Karim, Mamun S. Abdullah, Anisur M. Rahman, Ashraful M. Alam

**Affiliations:** Department of community Medicine, Abdul Malek Ukil Medical College (AMUMC), Begumgonj, Noakhali, 3820 Bangladesh; Filaria Control Program, CDC, Directorate General of Health Services (DGHS), Mohakhali, Dhaka, 1212 Bangladesh; Department of Epidemiology, National Institute of Preventive and Social Medicine (NIPSOM), Mohakhali, Dhaka, 1212 Bangladesh; Department of Maternal and Child Health, National Institute of Preventive and Social Medicine (NIPSOM), Mohakhali, Dhaka, 1212 Bangladesh

**Keywords:** Community clinic, Perceived quality, Satisfaction, Utilization

## Abstract

**Background:**

Bangladesh is one among the few countries of the world that provides free medical services at the community level through various public health facilities. It is now evident that, clients’ perceived quality of services and their expectations of service standards affect health service utilization to a great extent. The aim of the study was to develop and validate the measures for perception and satisfaction of primary health care quality in Bangladesh context and to identify their aspects on the utilization status of the Community Clinic services.

**Methods:**

This mixed method cross sectional survey was conducted from January to June 2012, in the catchment area of 12 community clinics. Since most of the outcome indicators focus mainly on women and children, women having children less than 2 years of age were randomly assigned and interviewed for the study purpose. Data were collected through FGD, Key informants interview and a pretested semi- structured questionnaire.

**Results:**

About 95 % of the respondents were Muslims and 5 % were Hindus. The average age of the respondents was 23.38 (SD 4.15) and almost all of them are home makers. The average monthly expenditure of their family was 95US $ (SD 32US$). At the beginning of the study, two psychometric research instruments; 24 items perceived quality of primary care services PQPCS scale (chronbach’s *α* = .89) and 22 items community clinic service satisfaction CCSS scale (chronbach’s *α* = .97), were constructed and validated. This study showed less educated, poor, landless mothers utilized the community clinic services more than their educated and wealthier counterpart. Women who lived in their own residence used the community clinic services more frequently than those who lived in a rental house. Perceptions concerning skill and competence of the health care provider and satisfaction indicating interpersonal communication and attitude of the care provider were important predictors for community clinic service utilization. Perception related to the quality of management, administration, physical environment of the service point and satisfaction addressing health promotion and women health issues played significant role on community clinic’s services utilization.

**Conclusions:**

Besides parental education and income, client’s perception and satisfaction played significant role in community clinic service utilization. Provider’s perception of service quality should be studied. The study findings will enable policy-makers to improve quality of primary health care services, realizing providers’ and patients’ ideas of community clinic service quality.

## Background

Bangladesh government has revitalized the community clinic project as a commitment of delivering free primary health care services at the doorstep of rural people and until now 12,527 independent community clinics have been made functional to deliver basic healthcare package to the community people, viz. maternal and child healthcare, reproductive health and family planning services, immunization, nutrition education, micronutrient supplementation, health education and counselling, communicable disease control, treatment for minor ailments and first-aid, and referral to higher-level health centres [1]. Community Clinics (CC) were to provide services for around 6000 people, and it was envisaged that their location would make them accessible for 80 % of the population within less than 30 min walking distance [2]. These Community Clinics were to bring family planning, preventive health services and limited curative services closer to the population, and to improve the efficiency of service provision, partly by replacing outreach services with services provided from a fixed point. Health Population Nutrition Sector Development Program (HPNSDP) has given much emphasis for its development and sustainability. It is now evident that services empathetically embrace the community accessibility and affordability issues let alone quality do not ensure its utilization and sustainability [3–6]. Experiences in Bangladesh [7] and also in China [8], Nepal [9] and other countries provide growing evidence that the perceived quality of health care service has a strong impact on their utilization status. The low utilization of both community health workers and first line health services is, to a large extent, due to consumers’ dissatisfaction and perceptions of low quality of care [10–17].

Satisfaction reflects the extent to which expectation of service standards have been met while perception of quality record patient rating about specific aspects of service quality [18–20]. The quality of care concept has been well-thought-out as a social phenomenon that vary across policy makers, professionals, managers, social workers, common users. This concept is also related to the type of care provided as well as to the social, physical and technical context in which the care is delivered [21]. As users’ viewpoints regarding service quality offers the potential to make services more responsive to people’s expectations, making health services better utilized, variety of approaches were attempted for a valid assessment of quality.

Researchers have developed a number of scales for measuring user’s perception and satisfaction with general practitioner services, where they identified different dimensions of primary care such as: access, office staff, privacy, waiting time, user’s own expectations, the competence and personal characteristics of the physician, empathy, listening, respect, provider skills, care coordination and environment. Andaleep et al compared several dimensions of perceived quality of care e.g. responsiveness, assurance, communication, discipline and “baksheesh”; unofficial payments, between public hospitals with private hospitals in Dhaka city and argued that these factors have a relatively greater influence on individuals’ decisions regarding utilization compared with access and costs. Haddad et al. developed and validated a 20 item scale in Guinea which is claimed to be an appropriate one for measuring lay peoples’ perception in a similar setting. Some of the studies use qualitative approaches with open interviews, while others use quantitative approaches based on structured questionnaires.

These measures differ in their dimensions, the number of items, the response formats, as well as the rules used to construct the global scores and some of the measures do not, however, provide sufficient methodological explanation [22–27]. Previous assessments of client satisfaction with services provided by government health workers in Bangladesh have mostly been limited to family planning services.

Health-care researchers who work with culturally diverse communities stated the importance that the measurement of quality related to primary health care services and satisfaction may vary in different cultural settings [26]. Bangladeshi cultural values may influence the measurement of service quality and primary care satisfaction; this study was conducted to determine whether the proposed scale structure of the primary care perception and satisfaction in its present form taps into these culturally salient values, and thus whether it is appropriate for use with Bangladeshi rural community [27].

The first objective of this study is to develop instruments to measure community perception and satisfaction regarding primary health service quality in Bangladesh and evaluate their reliability, validity. The second objective is to identify aspects of perceived quality and satisfaction which have large effects on the utilization status of the Community clinic services.

**Fig. 1:**
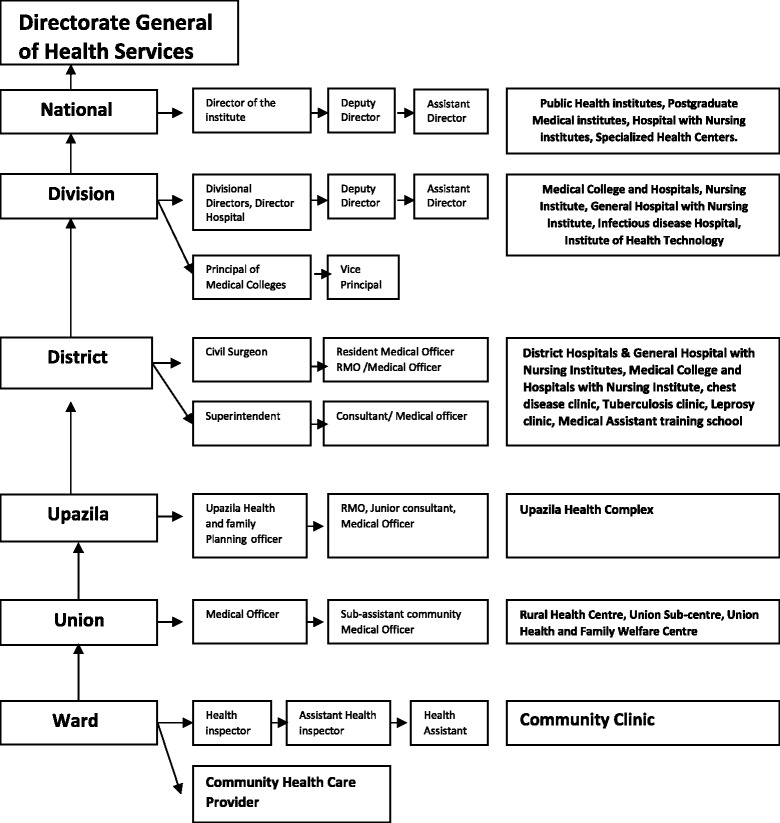
Types of health facilities from national toward level, with managerial hierarchy. Courtesy: Bangladesh health bulletin, 2013. Published Feb, 2014; P24

For better understanding of functional and managerial hierarchy of the Bangladeshi health system, an organogram (Fig. 1) is incorporated accordingly.

## Methods

This mixed method cross sectional survey was conducted from January to June 2012 in the catchment area of twelve community clinics, located at six districts. The respondents were enrolled following simple random sampling technique. Previous study showed 68.9 % patients expressed satisfaction with the provider’s usual behavior in primary healthcare settings of Bangladesh [28]. Assuming *p* = .689, 95 % confidence interval, 80 % power, 3 % margin of error, the estimated sample size was 915. The sample was further increased by 5 % to account for contingencies such as non-response or recording error, with a final figure of 960.

Bangladesh [Total 64 Districts]

 Random selection of 6 districts

6 Districts

 Random selection of one upazila from one district

6 Upazilas (Upazilas are basic unit of administration of the country. Upazilas are similar to the county subdivisions found in some western countries. Total no of upazilas are 509.)

 Random selection of one union from each Upazila

6 Unions (Union Councils are the smallest rural administrative and local government units in Bangladesh. Each Union is made up of nine Wards. There are 4550 Unions in Bangladesh.)

 Random selection of two wards from each union

12 Wards (a village or ward is the smallest territorial and social unit for administrative and representative purposes. At the 1991 census, villages in Bangladesh had an average of 232 households.)

 Random selection of eighty mothers having children less than two years of age from each ward

960 respondents

**Fig. 2:**
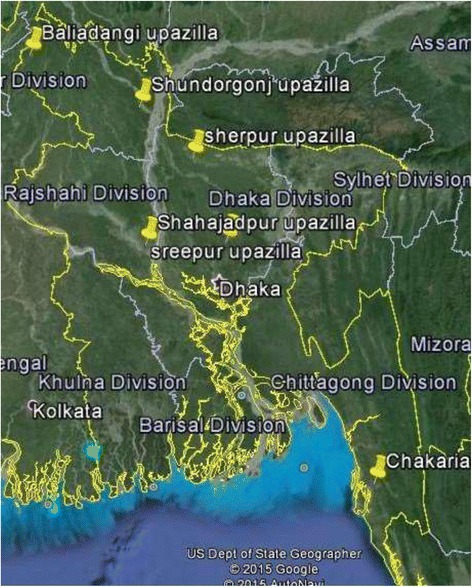
Map showing six underpinned Upazilla (subdistrict) selected at random for data Collection; Baliadangi, shundorgonj, Sherpur, Shahajadpur, Sreepur, Chakaria. [This map is reproduced unaltered from google map following their “uses in print” guideline]

Firstly six districts were chosen by lottery, from which six Upzilas (Sundargonj, Baliadangi, Ullapara, Sherpur, Sreepur and Chakaria) were selected randomly. (underpinned areas in Google Map, Fig. 2).

Random allocation of one union from each upazila, and two wards from each union, yielded 12 wards (each ward comprises approx. 6000 population). From each ward, 80 eligible mothers (Mothers having children less than 2 years of age and one mother from each household) were randomly assigned and interviewed for the study purpose. Since most of the rural males seldom stay at home during day time and most of the important outcome indicators focus mainly on women and children health, researcher deliberately enrolled women respondents for the study purpose.

After a five years’ closure, community clinics were reopened for the last two and half years and for this reason, it was logical to select mothers from those households having children aged less than 2 years, to investigate the utilization of selected health care services among them. Data were collected by six trained data collectors through face to face interview at the household level using interviewer administered semi-structured questionnaire. To cross check the information provided by the respondents regarding CC services related to the children and mother, relevant documents were also reviewed (Vaccination card, Pregnancy Card, Prescriptions, etc.). Community clinic services are multifaceted and inter-related operationally termed as essential service package (ESP), off which researcher preferred five of the most important amenities; “treatment for general illness or limited curative care”, “health education”, “maternal and child health counselling”, “family planning”, “antenatal and postnatal care” as outcome indicators for evaluating their utilization status. Written informed consents were taken prior interviewing the respondents. Ethical clearance was obtained from ethical review board of Bangladesh Medical Research Council.

### Development of the tool

An exploratory study was conducted at the first phase to develop and evaluate the properties of two new scales for measuring perceived quality and satisfaction related to primary health care services in Bangladesh. Slim Haddad’s 20-item scale for measuring perceived quality of primary health care services that includes three subscales related to health care delivery, personnel and facilities has been tested and validated in Guinea and Burkina Fuso. Researcher chose this measure as reference for developing a new tool [16, 18]. We conducted twelve homogenous focus group discussions (stratified by sex and age), 24 key informants interview of local people from six community clinic areas adjacent to the selected study areas to identify the criteria they use to judge the quality of service at community clinic. Their statements were recorded, translated into English, and transcribed under the supervision of experienced researchers. Comments and concepts revealing respondents’ views on quality were extracted. At the same time, different studies on patient’s perception of quality were meticulously reviewed to explore further scale items [12, 16, 18–20]. Some of the attributes were found common in both processes e.g. qualitative data extraction and literature review, such as; health provider’s skill in detecting health problem, quality of drugs supplied, treatment outcome, prescription quality, monitoring (late open and early closure, favoring relatives), equipment (necessary for clinical examination and primary laboratory examinations), availability of staffs, adequacy of the examination area, personal characteristic of the health worker (sympathetic, respectful, open hearted and honest), time given for explaining their illness and distance from their residence. Some attributes, e.g. what extent they can examine (female patient), counselling skill, information regarding tests and procedures, confidentiality, hurriedness of the health workers while attending clients, punctuality, bribe, hassle in collecting drugs and the physical environment (external environment, water supply, toilet facilities) were newly extracted. Results from both efforts led to an initial list of 30 quality attributes addressing skill and competence of the health care provider, management, administration and physical arrangement of Community Clinic.

In the second phase, a survey was undertaken to prioritize these criteria according to the degree of importance the users ascribe to them when judging quality of care. Sixty randomly selected respondents; ten from each ward of six selected unions were recruited for this purpose. The questionnaire was produced in the third phase. It was drafted simultaneously in English and Bengali, following a process of back translation. The back translation process was completed with the collaboration of “Health education and behavioral science” and “Community medicine” departments of National Institute of Preventive and Social Medicine (NIPSOM). The face validity of the questionnaire was openly assessed through feedback from a panel of experts (researchers, managers of community clinic revitalization program, specialists from MIS, DGHS and faculties of NIPSOM) who reviewed the questionnaire and confirmed it with minor wording changes. Content validity was revealed by asking experts to review the adequacy of the content of the instrument. They were asked to rate the clarity, the concreteness, the centrality, and the importance of each item. Inter-rater agreement was analyzed for every item to value their adequacy.

The questionnaire was then pretested on 24 people of the adjacent communities of the selected areas to allow for adjustment of wording. Each question refers to one of the 30 attributes identified in the previous phase. For each question, respondents could express one of four opinions: do not know (0), not good (1), average (2), or good (3). The scoring system differs with that of Haddad et al as we scored “do not know” = 0 and “not good” =1 assuming the fact that those who never took any of the community clinic services, should have lower score than those who at least went to community clinic for some purpose though their perception regarding those services was not favorable.

### Statistical analysis

The data was entered, cleaned and edited with the help of Software “Statistical Package for the Social Sciences” (SPSS) for windows version 20.0. After describing the data, chi-square and Student’s t test were performed to determine the association between socio-demographic characteristics, PQPCS, CCSS and utilization of CC services. Finally, to predict the role of perceived service quality and satisfaction of service utilization, while controlling for possible confounders, those variables showed *p* < .20 in univariate analysis, were included in binary logistic regression models. Adjusted Odds ratios (AOR), 95 % confidence intervals (95 % CI) and *p* values were calculated for each potential contributors. Cox and Snell and Negelkerke R^2^ referring the explained variability and ROC values with 95 % CIs indicating case classification status of the model were also reported.

## Results

### Construction of scales and subscales for measuring quality

#### Factor analysis [Table 1]

**Table 1:** Factor analysis for perceived quality of primary health care PQPCS

		Component				Corrected Item-Total Correlation	Cronbach’s Alpha if Item Deleted	Eigen values (% of variance)	Cronbach’s Alpha
		1	2	3	4				
Skill and competence (12 items)									
1	Counseling skill	.849				.493	.875	7.41(24.61)	.90
2	Respectfulness	.835				.551	.872		
3	Skill in detecting health problem	.801				.536	.874		
4	Confidentiality	.745				.407	.877		
5	What extent they examine	.733				.675	.869		
6	Sympathetic	.660				.717	.869		
7	Honesty	.637				.378	.879		
8	Time spent explaining female patient	.633				.746	.868		
9	Hurry during examining patient	.616				.543	.873		
10	Open hearted	.562				.733	.868		
11	Information on test &procedures	.459				.449	.876		
12	How good their prescription	.451				.237	.880		
Management (5 items)									
13	Collecting drugs from cc		.840			.491	.875	3.68(15.26)	.80
14	Monitoring		.823			.250	.885		
15	Overall management		.649			.571	.872		
16	Time spent explaining their illness treatment		.585			.708	.869		
17	Result of treatment		.572			.351	.878		
Administration									
18	Staff available			.806		.481	.875	1.87(11.13)	.76
19	Examining place(area used)			.743		.236	.881		
20	Health provider available			.660		.486	.875		
21	Timeliness/punctuality			.626		.295	.879		
Physical environment									
22	Toilet facilities				.836	.348	.879	1.62(9.75)	.74
23	Drinking water supply				.818	.180	.885		
24	External environment (cleanliness)				.569	.423	.876		

Total variance = 60.74; Cronbach’s Alpha = .89 (24 items)

The 30 items of Perceived quality primary care service PQPCS measure were subjected to principal components analysis (PCA) using SPSS Version 20. Prior to performing PCA the suitability of data for factor analysis was assessed. Inspection of the correlation matrix revealed the presence of many coefficients of .3 and above. The Kaiser-Meyer-Oklin value was .89, exceeding the recommended value of .6 [29, 30] and the Barlett’s Test of Sphericity [31] reached statistical significance, supporting the factorability of the correlation matrix. Principal components analysis revealed the presence of four components with eigenvalues exceeding 1, explaining 30.9, 15.3, 7.8 and 6.8 % of the variance respectively. An inspection of the scree plot revealed a clear break after the 4th component. Using Cattell's [32] scree test, it was decided to retain four components for further investigation. This was further supported by the results of Parallel Analysis, which showed only four components with eigenvalues exceeding the corresponding criterion values for a randomly generated data matrix of the same size (30 variables × 960 respondents).

To aid in the interpretation of these four components, Varimax rotation was performed. The rotated solution revealed the presence 24 items of four components showing a number of strong loadings (> .3) and loading substantially on only one component (ranged.45-.84). The four component solution explained a total of 60.7 % of the variance, with Component 1 = 24.6 %, Component 2 = 15.3 %, component 3 = 11.1 % and factor 4 contributing 9.75 %. Thus, the perceived quality primary care service PQPCS scale was formed from four dimension and 24 items. The first group (α) included twelve items related to the attitudes and practices of the health care workers: counselling skill, respectfulness, diagnostic skill, confidentiality, adequacy and extent of clinical examination, sympathy, honesty, open heartedness, information about test and procedures, time spent for explaining women health problem, and lastly, prescribing efficiency. The second group included five items referring to management; collecting drug, monitoring, overall management, IEC material and quality of drugs. The four items in the third group focused more specifically on administrative procedure; staff availability, examining place, timeliness and punctuality of the caregiver. Three items of fourth group referred to physical facilities; toilet facilities, drinking water supply, cleanliness and external environment. It is important to be noted that items related to fees, distance, drug quality, bribe, equipment are dropped down from the final scale and at the same time items related to counselling skill, confidentiality, punctuality and physical environment are included. A comparison of the newly develop 24 item PQPCS scale derive from initial 30 scale items with Haddad’s 20 item perception of primary health care service quality scale is shown in Appendix.

### Internal consistency

The analysis revealed four factors; attitudes and practices of the health care workers, management, administrative procedure, physical arrangement of CC. The PQPCS was found to have an overall coefficient alpha of 0.89. Alphas of the four factors ranged from 0.74 to 0.90 (see Table 1). The corrected item total correlations for the remaining 24 items ranged from 0.24 to 0.74 except one (drinking water supply; .18).

### Construction of scales and subscales for measuring satisfaction

The community clinic service satisfaction (CCSS) scale was developed complying with the same basic principles as followed in constructing PQPCS scale. The primary care satisfaction survey for women (PCSSW) developed by Scholle and colleagues 2004; is a 24-item survey tool consisting of three subscales that have been shown to be psychometrically valid among Turkish women was chosen as a reference tool for the purpose [26] Each PCSSW item is rated on a 5-point scale: 1 = not at all satisfied; 2 = somewhat satisfied; 3 = satisfied; 4 = very satisfied; and 5 = extremely satisfied. As no study found to be focused specifically on PCSSW, there might be reason to believe that the measurement of the primary care satisfaction may differ in different cultural context. Therefore, we planned to validate the scale items of PCSSW to make it representative of the constructs of community clinic service satisfaction CCSS scale from Bangladesh perspective and to customize it as culturally sensitive.

### Factor analysis [Table 2]

**Table 2:** Factor analysis for community clinic service satisfaction CCSS

		Component			Corrected Item-Total Correlation	Eigen value Variance)	Cronbach’s Alpha
		1	2	3			
Interpersonal skill and attitude of the care provider							
1	The amount of time I had to talk with my health professional	.814			.740	12.9(32.94)	.95
2	The staff ’s flexibility in scheduling my appointment around my needs	.802			.791		
3	The courtesy of the staff	.797			.730		
4	My health professional’s ability to answer questions in a sensitive and caring way	.783			.816		
5	My health professional’s ability to help me feel comfortable talking about my concerns	.768			.847		
6	The chance to ask all of my questions	.762			.806		
7	My overall trust in the health professionals here	.715			.716		
8	Privacy when talking to the receptionist	.706			.676		
9	My health professional’s ability to take what I say seriously	.663			.836		
10	My health professional’s willingness to explain different options for my care	.607			.819		
11	How well the staff kept you informed about the waiting time	.448			.605		
Preventive and promotive health services							
12	How well the health professionals explain the results of tests or procedures		.813		. .722	1.7(23.25)	.92
13	Information about how to get the results of my tests		.791		.765		
14	The health professionals’ focus on prevention		.684		.468		
15	How well my health care fits my stage of life		.684		.775		
16	The health professionals’ interest in my mental and emotional health		.666		.774		
17	The information I get about healthy living. (such as diet and exercise		.630		.776		
18	My health professional’s interest in how my life affects my health		.622		.787		
Women health issue							
19	The health professionals’ knowledge of women’s health issues			.819	.664	1.1(14.97)	.86
20	Help with finding information resources in women’s health			.755	.663		
21	My health professional’s ability to explain things clearly			.625	.747		
22	The chance to get both gynaecological and general health care here			.570	.675		

Total variance = 71.16; Cronbach’s *α* = .97 (22 items)

A factor analysis of the current results was performed using the Maximum Likelihood method of extraction. Bartlett’s test of sphericity was significant [χ^2^ (231) = 18916.51, *p* < 0.001], and Kaiser-Meyer-Olkin measure of sampling adequacy was .97indicating that it was appropriate to use the factor analytic model on this set of data. PCA revealed the presence of 3 factors with eigenvalues greater than one which indicated that three factors gave the most interpretable solution. Using Cattell’s [32] scree test, it was decided to retain three components for further investigation. An Oblimin rotation was performed since factors were expected to be correlated. The obtained pattern matrix is displayed in Table. Only 22 items with factor loadings of above .35 are shown. (Two items of PCWSS were dropped as they showed smaller loading < .3” help me scheduling my next visit”,” the chance to talk with my health professionals with my clothes on”). The first factor was robust, with a high eigen value of 12.95, and it accounted for 58.84 % of the variance in the data. Factor two had an eigenvalue of 1.7 and accounted for a further 7.76 % of the variance. The eigenvalues for factors three were 1.1 accounting for a further 4.6 % of the total variance.

The pattern matrix revealed factor one to consist of eleven items. This factor was labelled interpersonal skill and attitude of the care provider and demonstrated a high internal consistency (chronbach’s *α* = .95). The second factor consisted of seven items including preventive and promotive health services questions and reflected a high internal consistency (chronbach’s *α* = .92). Factor three contained four items relating to the issues of women health and was labeled women health issues. The internal consistency of this item was also high (chronbach’s *α* = .86). In summary, the three factors retained were interpersonal skill and attitude of the care provider, preventive and promotive health services, women health issues. These three factors were considered subscales of community clinic service satisfaction (CCSS) scale for Bangladeshi women amenable to measure women’s related to community clinic services.

### Internal consistency

The analysis revealed three factors; interpersonal skill and attitude of the care provider, preventive and promotive health service, women health service of CC. The CCSS was found to have an overall coefficient alpha of 0.97. Alphas of the three factors ranged from 0.86 to 0.95 (see Table).

### Role of perceived quality and satisfaction on the Community clinic service utilization

To identify aspects of perceived quality and satisfaction on the utilization status of the Community clinic services, 960 respondents were interviewed with the newly developed tools from 12 community clinic catchment areas. The mean (SD) age of the respondents was 23.4 (4.15) and almost all of them are home makers. About 95 % of the respondents were Muslims and 5 % were Hindus. The average monthly expenditure of their family was 7462.92TK (SD 2545 TK) that is equivalent to 95US $ (SD 32US$). Detail description of the sociodemographic characteristics are presented in Table 3. Study result showed limited curative care service utilization provided by community clinics was significantly associated with mother’s education, age, education, occupation of father, average monthly family income, residential and cultivable land ownership. These explanatory variables also found significantly associated with the health education service delivered by the selected community clinics. Limited curative care was found related to all domains of PQPCS and interpersonal skill and attitude of the care provider domain and women health issue domain of CCSS but health education revealed significant association with all domains of both PQPCS and CCSS. Maternal and child health counselling services provided by the community clinic showed significant relationship with parental characteristics along with cultivable land ownership, latrine use and availability of electricity. Counselling was also found associated with three domains (Skill and competence, Management, Administration) of PQPCS and all domains of CCSS. Collection of family planning material was not applicable for all samples. Analyzing the eligible subsample data, parental age, their educational attainment, husband’s occupational status, availability of electricity, all domains of both PQPCS and CCSS revealed significant relationship with FP material collection from CC. Parental characteristics (age, education, occupation), family income, land used for residence and type of latrine showed significant association with the ANC and PNC services provided by the CCs. Three domains (Skill and competence, management, physical environment) of PQPCS and one domain (women health issue) of CCSS were also found related to ANC and PNC services [Table 3].

**Table 3:** Univariate analysis between Sociodemographic characteristics, PQPCS, CCSS subscales with service utilization status of Community Clinic

Characteristics	Limited curative care			Health education			Counselling service(MCH)			FP material*			ANC & PNC		
	No/n (%)	Yes/n(%)	_χ_2	No/n (%)	Yes/n(%)	_χ_2	No/n (%)	Yes/n(%)	_χ_2	No/n (%)	Yes/n(%)	_χ_2	No/n (%)	Yes/n(%)	_χ_2
			(*P* value)			*P* value			*P* value			*P* value			*P* value
Maternal age															
<20	49(35)	90(65)	NS	87(63)	52(37)	2.83	71(51)	68(49)	2.41	41(62)	25(38)	7.37	97(70)	42(30)	20.48
20-25	183(37)	312(63)		329(67)	166(33)	(.24)	272(55)	223(45)	(.29)	181(54)	152(46)	(.025)	404(82)	91(18)	(.000)
>25	133(41)	193(59)		229(70)	97(30)		191(59)	135(41)		90(45)	110(55)		285(87)	41(13)	
Maternal education															
No education	125(54)	107(46)	35.07	181(78)	51(22)	20.79	146(63)	86(37)	11.80	55(44)	69(56)	6.39	219(94)	13(6)	66.85
Preprimary	44(29)	110(71)	(.000)	88(57)	66(43)	(.000)	88(57)	66(43)	(.008)	48(53)	42(47)	(.09)	95(62)	59(38)	(.000)
Primary	158(35)	292(65)		291(65)	159(35)		245(54)	205(46)		162(52)	148(48)		370(82)	80(18)	
Secondary	38(31)	86(69)		85(69)	39(31)		55(44)	69(56)		47(63)	28(37)		102(82)	22(18)	
Husbands age															
<25	35(23)	119(77)	21.57	84(55)	70(45)	23.20	77(50)	77(50)	11.12	65(64)	36(36)	12.24	104(68)	50(32)	29.21
25-30	175(38)	283(62)	(.000)	298(65)	160(35)	(.000)	239(52)	219(48)	(.004)	157(54)	135(46)	(.002)	377(82)	81(18)	(.000)
>30	155(45)	193(55)		263(76)	85(24)		218(63)	130(37)		90(44)	116(56)		305(88)	43(12)	
Husbands education															
No education	174(60)	116(40)	90.45	259(89)	31(11)	98.86	202(70)	88(30)	37.14	51(37)	88(63)	18.44	282(97)	8(3)	92.03
Preprimary	31(21)	116(79)	(.000)	74(50)	73(50)	(.000)	83(37)	64(43)	(.000)	51(53)	46(47)	(.000)	94(64)	53(36)	(.000)
Primary	111(29)	267(71)		224(59)	154(41)		183(48)	195(52)		153(57)	116(43)		284(75)	94(25)	
Secondary	49(34)	96(66)		88(61)	57(39)		66(45)	79(55)		57(61)	37(39)		126(87)	19(13)	
Occupation of husband															
Farmer	92(32)	197(68)	8.50	174(60)	115(40)	9.44	161(56)	128(44)	22.26	78(48)	85(52)	8.25	216(75)	73(25)	14.45
Daily labor	112(41)	163(59)	(.037)	195(70)	80(29)	(.024)	181(66)	94(34)	(.000)	78(48)	83(52)	(.04)	236(86)	39(14)	(.002)
Service	124(43)	167(57)		201(69)	90(30)		148(51)	143(49)		118(61)	77(39)		246(85)	45(15)	
Skilled labor	37(35)	68(65)		75(71)	30(29)		44(42)	61(58)		38(48)	42(52)		88(84)	17(16)	
Family income															
<5000	24(20)	94(80)	24.46	60(51)	58(49)	45.91	58(49)	60(51)	2.45	48(61)	31(39)	3.43	69(59)	49(41)	60.16
5000-10000	242(38)	394(62)	(.000)	410(65)	226(35)	(.000)	357(56)	279(44)	(.29)	197(50)	199(50)	(.18)	526(83)	110(17)	(.000)
>10000	99(48)	107(52)		175(85)	31(15)		119(58)	87(42)		67(54)	57(46)		191(93)	19(7)	
House ownership															
Rental	17(38)	28(62)	NS	25(56)	20(44)	2.90	22(49)	23(51)	.86	9(39)	14(61)	1.61	28(62)	17(38)	12.29
Own house	348(38)	567(62)		620(68)	295(32)	(.104)	512(56)	403(44)	(.22)	303(53)	273(47)	(.28)	758(83)	157(17)	(.000)
Residence land															
<5	62(26)	174(74)	40.61	139(59)	97(41)	42.60	137(58)	99(42)	.86	78(52)	73(48)	.55	165(70)	71(30)	33.22
5-10	119(33)	239(67)	(.000)	214(60)	144(40)	(.000)	194(54)	164(46)	(.65)	124(54)	106(46)	(.7)	298(83)	60(17)	(.000)
>10	184(50)	182(50)		292(80)	74(20)		203(56)	163(44)		110(51)	108(49)		323(88)	43(12)	
Cultivable land															
0 decimal	200(41)	289(59)	7.29	343(70)	146(30)	3.95	290(59)	199(41)	5.88	147(51)	144(49)	2.01	404(83)	85(17)	2.79
<50 decimal	44(29)	109(71)	(.026)	98(64)	55(36)	(.14)	76(50)	77(50)	(.05)	64(58)	46(42)	(.37)	118(77)	35(23)	(.23)
= > 50 decimal	121(38)	197(62)		204(64)	114(36)		168(53)	150(47)		101(51)	97(49)		264(84)	54(16)	
Type of latrine															
Pit type	194(40)	288(60)	2.04	338(70)	144(30)	3.79	298(62)	184(38)	15.08	157(52)	145(48)	NS	433(90)	49(10)	41.32
Sanitary	171(36)	307(64)	(.16)	307(64)	171(36)	(.05)	236(49)	242(51)	(.000)	155(52)	142(48)		353(74)	125(26)	(.000)
Electricity															
No	260(40)	390(60)	3.35 (.075)	429(66)	221(34)	1.29	393(61)	257(39)	19.08	179(46)	209(54)	15.64	524(81)	126(19)	2.15
Yes	105(34)	205(66)		216(70)	94(30)	(.27)	141(44)	169(56)	(.000)	133(63)	78(37)	(.000)	262(85)	48(15)	(.14)
Scales and subscales	Mean(SD)		t (*p* value)	Mean(SD)		t (*p* value)	Mean(SD)		t (*p* value)	Mean(SD)		t (*p* value)	Mean(SD)		t (*p* value)
PQPCS															
12 item (name of subscale)	19.63(4.95)	22.13(4.41)	-7.92(.000)	20.35(4.77)	22.89(4.34)	-8.25(.000)	19.38(4.80)	23.44(3.66)	-14.87(.000)	20.62(4.71)	22.03(4.62)	-3.7(.000)	20.85(4.85)	22.67(4.15)	-5(.000)
5 item	10.19(2.31)	8.79(2.36)	8.99(.000)	9.84(2.48)	8.27(1.96)	10.63(.000)	9.07(2.26)	9.65(2.61)	-3.66(.000)	8.38(2.21)	9.66(2.42)	-6.75(.000)	9.49(2.46)	8.56(2.17)	5.03(.000)
4 item	6.33(1.78)	5.73(1.10)	5.76(.000)	6.08(1.59)	5.30(.98)	4.55(.000)	5.64(1.07)	6.35(1.70)	-7.58(.000)	5.57(.93)	5.90(1.35)	-3.44(.001)	5.98(1.50)	5.85(1.04)	1.36(.17)
3 item	4.88(1.72)	4.66(1.41)	2.05(.04)	4.89(1.61)	4.44(1.32)	4.65(.000)	4.66(1.49)	4.85(1.59)	-1.86(.06)	4.41(1.41)	5(1.51)	-4.90(.000)	4.86(1.58)	4.22(1.22)	5.95(.000)
PCWSS															
11 item	21.04(7.58)	22.76(6.81)	3.54(.000)	21.20(6.67)	23.95(7.77)	-5.39(.000)	19.41(5.90)	25.48(7.17)	-14.07(.000)	20.39(6.66)	23.42(7.08)	-5.39(.000)	21.29(6.62)	25.80(8.27)	-6.74(.000)
7 item	11.28(4.11)	11.17(3.54)	.43(ns)	10.99(3.82)	11.67(3.63)	-2.68(.008)	9.75(2.95)	13.05(3.88)	-14.53(.000)	10.34(3.44)	11.71(3.5)	-4.84(.000)	11.18(3.72)	11.35(3.99)	-.51(.61)
4 item	9.30(2.82)	10.15(2.37)	4.85(.000)	9.48(2.58)	10.55(2.43)	-6.31(.000)	8.95(2.38)	10.93(2.40)	-12.78(.000)	9.24(2.53)	10.48(2.38)	-6.19(.000)	9.76(2.51)	10.13(2.87)	-1.57(.12)

* mark signifies that the total number of respondent (599) who took the different family planning services from community clinics are less than the total number of respondents in the study (960) because all mothers are not eligible for that sevice

To identify the important predictors and to control the confounding effects of other variables, five binary logistic regression models were constructed. Model characteristics indicating R^2^ and ROC cut-offs with 95 % CIs are presented in the Table 4. First model showed educational status of mother, father’s occupation, average monthly family income, residential and cultivable land ownership increased the likelihood of curative care service utilization provided by community clinics. In this study, mother’s literacy (pre-primary education), their residence ownership, perceived quality related to health carer skill and competence, satisfaction related to their interpersonal skill and attitude showed increased probability of limited curative care services to be utilized. On the other hand, father’s service, their solvency and land ownership, their perception related to community clinic management quality, satisfaction related to preventive and promotive health services decreased the likelihood of curative care service utilization. Second model revealed, husband’s education and perceived quality related to health carer’s skill and competence found significantly increased the chance of health-education service utilization delivered by the selected community clinics. On the contrary high income, perceived quality related to management, perceived quality related to physical environment decreased the chance to seek this service. Limited curative care was found related to two domains of PQPCS and 1st and third domain of CCSS whereas health education revealed significant association with all domains of PQPCS except administrative quality domain but not related with any of the CCSS subscales.

**Table 4:** Binary logistic regression models for identifying significant predictors of Community Clinic service utilization status

Predictors	Model 1:General Health/Limited curative care	Predictors	Model 2: Health information	Predictors	Model 3: Counselling (MCH)	Predictors	Model 4: Collection of FP material	Predictors	Model 5: ANC and PNC services
	χ^2^ (*p* value)		χ^2^ (*p* value)		χ^2^ (*p* value)		χ^2^ (*p* value)		χ^2^ (*p* value)
	[AOR with (95 % CI)]		[AOR with (95 % CI)]		[AOR with (95 % CI)]		[AOR with (95 % CI)]		[AOR with (95 % CI)]
Mothers with preprimary education	4.20(.04) (.043) [1.89(1.03, 3.53)]	Husband’s preprimary education	8.41(.004) [3.03(1.43, 6.41)]	Husband’s primary education	4.68(.03) [1.81(1.06, 3.10)]	Average monthly family income 5000-10000	10.93(.001) [3.64 (1.69, 7.82)]	Mothers age more than 25 years	4.11 [.330 (.11, .96)]
Husband occupation [Service]	4.76(.029) [.56(.33, .94)]	Husband’s preprimary education	9.10(.003) [2.77(1.43, 5.38)]	Husband’s secondary education	5.23(.022) [2.28 (1.13, 4.60)]	Average monthly family income >10000	3.93(.047) [2.59 (1.01, 6.65)]	Mothers completed secondary education	5.51(.019) [3.40 (1.22, 9.46)]
Average monthly family income 5000-10000	8.83(.003) [.37(.19, .71)]	Husband’s secondary education	9.18(.002) [3.56(1.57, 8.09)]	Perceived quality related to Skill and competence	38.87(.000) [1. 24 (1.16, 1.32)]	Sanitary latrine users	5.16(.023) [1.78 (1.08, 2.94)]	Husband’s preprimary education	12.60(.000) [5.93 (2.22, 15.82)]
Average monthly family income >10000	5.02(.025) [.40(.18, .89)]	Average monthly family income >10000	4.47(.034) [.40(.17, .94)]	Perceived quality related to management	10.97(.001) [.84 (.76, .93)]	Perceived quality related to management	6.22(.013) [1.17 (1.03, 1.32)]	Husband’s primary education	11.57(.001) [4.87 (1.96, 12.12)]
Own houses	7.67(.006) 3.23(1.41, 7.40)	Perceived quality related to to Skill and competence	24.00(.000) [1.21(1.12, 1.30)]	Perceived quality related to administration	27.49(.000) [1.61(1.35, 1,93)]	Satisfaction related to women health issue	3.57(.05) [1.16 (1.00, 1.36)]	Cultivable land = > 50 decimal	5.01(.025) [.48 (.25, .91)]
Residential land 5-10 decimal	5.51(.019) [.56(.35, .91)]	Perceived quality related to management	37.10(.000) [.69(.61, .78)]	Satisfaction related to interpersonal skill and attitude of the care provider	7.42(.006) [1.08(1.02. 1.14)]			Perceived quality related to management	10.59(.001) [.79 (.68, .91)]
Residential land >10 decimal	6.70(.010) .50(.29, .84)	Perceived quality related to physical environment	9.27(.002) [.781(.67, .92)]	Satisfaction related to preventive and promotive health services	4.23(.040) [1.09(1.01, 1.19)]			Perceived quality related to administration	4.87(.027) [.77 (.61, .97)]
Perceived quality related to Skill and competence	16.90(.000) 1.14(1.07, 1.22)							Satisfaction related to preventive and promotive health services	3.74(.053) [.90 (.81, 1.00)]
Perceived quality related to management	34.45(.000) .73(.65, .81)								
Satisfaction related to interpersonal skill and attitude of the care provider	7.07(.008) 1.081(1.02, 1.15)								
Satisfaction related to preventive and promotive health services	5.10(.024) .90(.82, .99)								
Cox & Snell/Nagelkerke R^2^ ROC (95 % CI)	.36/.49 .87 (.85, .90)		.37/.52 .88 (.86, .90)		.35/.47 .85(.83, .88)		.31/.41 .83(.79, .86)		.29/.48 .86 (.83, .9)

*AOR* Adjusted Odds Ratio, *CI* Confidence Interval, *ROC* Receiver Operating Characteristic Curve

Third model indicated husbands’ educational attainment, perception referring to Skill and competence of the care provider (PQPCS 1st domain), administration (domain3 of PQPCS), satisfaction indicating interpersonal skill and attitude, preventive and promotive health enhanced the likelihood of getting maternal and child care counselling services whereas perceived quality related to management decreased the possibility of getting this service. Model four presented that higher income status, families having good sanitation, perception referring to better management of CCs and satisfaction addressing women health related issues raised the probability of collecting family planning material from CCs. Lastly, in model five, maternal age (>25 years), higher education of spouses, families having less cultivable land, showed elevated chance of ANC and PNC service utilization, while lower utilization of this service was subjected to perceived quality related to management administration, and satisfaction related to preventive and promotive health services [Table 4].

## Discussion

Relying on the existing literature reviewed this study is assumed to be a cogent attempt to assess the role of consumer’s perception of quality and satisfaction on CCs service utilization status. The primary challenges we met were to construct appropriate tools for measuring these two psychometric issues which needed to be sensitive as well as specific in a primary care setting. Accordingly, researchers constructed a 24 item PQPCS scale that included four unique domains; perception referring to skill and competence of the care provider, management quality, administrative adequacy and physical environment of community clinics. The detail of the scale components and the validity issues have been discussed in earlier section of this article. The 22 items community clinic service satisfaction CCSS scale was constructed from 24 items PCWSS by Scholle and colleagues, 2004. In this study CCSS items were clustered in three domains; interpersonal skill and attitude of the care provider, preventive and promotive health and women health related issues.

Community clinics are one stop service centers which were set to deliver all of primary health care in their catchment areas. This study revealed that poverty, women education, occupation and education of the husband, landownership significantly alter the likelihood of service utilization status. Perception referring skill and competence of the care provider and satisfaction relating interpersonal skill and attitude of the care provider and in some occasions, perception regarding management and administration, satisfaction indicating preventive and promotive health and women health related issues played significant role on community clinic service utilization. Stratified data also showed that utilization varied significantly among the selected areas. The variation of skill and competency of health worker, physical environment of the settings, socio demographic diversity might explain this variation.

The overall services of CC depend not only on skill and coordination of these personnel but also on regular drug and equipment supplies, training, monitoring and support from higher authorities.

The process used to identify the scale content is inductive, and is designed to focus on the concerns and visions of the lay people, which will obviously differ from the concept of quality held by researchers, health care authorities and providers. The unexplained variability found in these models, might also be due to the contributions of providers’ perception and satisfaction related to the services they are providing.

Another limitation of the study could be that only women having children aged 2 years were included in this study; might limit generalization to the population. In this study, majority of patients were found satisfied, might reflect a low expectation level owing to their lifelong experience of spending a short time with health care providers. This study showed that the perceived technical quality of care for the client plays a lesser role in affecting utilization than the interpersonal nature of care.

## Conclusions

The PQPCS and CCSS scales for measuring perception and satisfaction were developed and validated complying adequate methodological issues. This study confirms findings in developing countries that the perception and judgement of quality are highly individualistic and dynamic, in the sense that the criteria or elements used for judging quality change with time and context. Apart from socioeconomic characteristics, perceptions referring skill and competence, management and administrative qualities of the CCs, satisfaction indicating interpersonal skill and attitude of the care provider, health education and women health related issues presented significant influences on community clinic service utilization.

## Abbreviations

BMRC, Bangladesh medical research council; CC, community clinic; CCSS, community clinic service satisfaction; DGHS, Directorate General of Health Services; ESP, Essential Service Package; HPNSDP, Health Population Nutrition Sector Development Program; NIPSOM, National Institute of Preventive and Social Medicine; PQPCS, perceived quality of primary care services.

## Appendix

**Table 5:** Perceived quality primary care service scale PQPCS was formed from initial 30 items (extracted from community interview, FGD, literature search) which now finally includes four dimensions and 24 items

Slim Haddad’s 20 scale items for measuring health service quality	Initial 30 attributes stated by lay people to judge quality	Final scale items resulted after factor analysis
In your opinion, are the “doctors” in the “hospital” capable of finding out what is wrong with the patients?	Skill in detecting health problem	Skill in detecting health problem
In your opinion, are the drugs needed that the “doctors” in the hospital prescribe to patients…	How good their prescription	How good their prescription
In your opinion, patients can obtain drugs easily from this hospital	Collecting drugs from cc	Collecting drugs from cc
The drugs supplied by this “hospital are good	Quality of drug	X
The patients cared for in this hospital recover good	Result of treatment	Result of treatment
In your opinion, the “doctors” in the hospital examine their patients well	What extent they examine	What extent they examine
In your opinion, the “doctors” in the hospital monitor their patients’ recovery well	Monitoring	Monitoring
In your opinion, the doctors in the hospital are open with the patients	Open hearted	Open hearted
In your opinion, the “doctors” in the “hospital” are compassionate towards the patients.	Sympathetic/compassionate	Sympathetic/compassionate
In your opinion, the “doctors” are respectful towards the patients	Respectfulness	Respectfulness
In your opinion, the time that the “doctors” devote to their patients is adequate	Hurry during examining patient	Hurry during examining patient
In your opinion, the time that the “doctors” take to explain to their patients about their illness is adequate	Time spent explaining their illness	Time spent explaining their illness treatment
In your opinion, the people who work in this “hospital” are honest	Honesty	Honesty
In your opinion, the fees that are charged in this “hospital” are reasonable	Rationality of free service	X
In your opinion, in this “hospital” patients have access to credit easily!	Total expenses	X
The distance from your home to the “hospital”… is reasonable	Distance community clinic	X
In your opinion, the number of “doctors” in this “hospital” is adequate	Health provider available	Health provider available
In your opinion, the “doctors” in the “hospital” are well suited to treat women’s diseases	Time spent explaining female patient	Time spent explaining female patient
In your opinion, the equipment in the “hospital” adequate for detecting diseases	Equipment in CC X	X
In your opinion, the waiting rooms, examination rooms, and “hospital” rooms are adequate	Examining place (area used adequate)	Examining place (area used adequate)
X	Counselling skill	Counselling skill
X	Information on test	Information on test
	&procedures	&procedures
X	Confidentiality	Confidentiality
X	Bribe	X
X	Timeliness/punctuality	Timeliness/punctuality
X	Staff available	Staff available
X	Toilet facilities	Toilet facilities
X	Drinking water supply	Drinking water supply
X	External environment	External environment
X	Overall management	Overall management
